# Dutch juvenile idiopathic arthritis patients, carers and clinicians create a research agenda together following the James Lind Alliance method: a study protocol

**DOI:** 10.1186/s12969-018-0276-3

**Published:** 2018-09-15

**Authors:** Casper G. Schoemaker, Wineke Armbrust, Joost F. Swart, Sebastiaan J. Vastert, Jorg van Loosdregt, Anouk Verwoerd, Caroline Whiting, Katherine Cowan, Wendy Olsder, Els Versluis, Rens van Vliet, Marlous J. Fernhout, Sanne L. Bookelman, Jeannette Cappon, J. Merlijn van den Berg, Ellen Schatorjé, Petra C. E. Hissink Muller, Sylvia Kamphuis, Joke de Boer, Otto T. H. M. Lelieveld, Janjaap van der Net, Karin R. Jongsma, Annemiek van Rensen, Christine Dedding, Nico M. Wulffraat

**Affiliations:** 1 Pediatric Rheumatology and Immunology, Wilhelmina Children’s Hospital, University Medical Center Utrecht, Utrecht, Netherlands; 2 Netherlands JIA Patient and Parent Organisation, member of ENCA, Amsterdam, The Netherlands; 3 National Institute for Public Health and the Environment (RIVM), Bilthoven, The Netherlands; 4 University Medical Center Groningen (UMCG), Beatrix Childrens Hospital, Dept Pediatric Rheumatology and Immunology, University of Groningen, Groningen, The Netherlands; 5 Dutch Association for Pediatric Rheumatology, Amsterdam, The Netherlands; 6 Faculty of Medicine, Utrecht University, Utrecht, The Netherlands; 7 James Lind Alliance, National Institute for Health Research Evaluation, Trials and Studies Coordinating Centre (NETSCC), based at the University of Southampton, Southampton, UK; 8 Youth-R-Well.com, Young Patient Organisation, The Netherlands, member of EULAR PARE, Amsterdam, The Netherlands; 9 Reade, Centre for Rehabilitation and Rheumatology, Department Rehabilitation, Amsterdam, The Netherlands; 10 Dutch Health Professionals in Pediatric Rheumatology (DHPPR), Amsterdam, The Netherlands; 11 Paediatric rheumatology, Emma Children’s Hospital, University Medical Centre Amsterdam, Amsterdam, The Netherlands; 12 Paediatric Rheumatology, Amalia Children’s Hospital, Radboudumc, Nijmegen, The Netherlands; 13 Paediatric Rheumatology, St. Maartenskliniek, Nijmegen, The Netherlands; 14 Paediatric Rheumatology, Leiden University Medical Centre, Leiden, The Netherlands; 15 Paediatric Rheumatology, Sophia Children’s Hospital, Erasmus University Medical Centre, Rotterdam, The Netherlands; 16 Department of Ophthalmology, University Medical Centre Utrecht, Utrecht, The Netherlands; 17 University Medical Center Groningen, Center for Rehabilitation, University of Groningen, Groningen, The Netherlands; 18 Child Development and Exercise Center, Division of Pediatrics. Wilhelmina Children’s Hospital, University Medical Centre Utrecht, Utrecht, The Netherlands; 19 Julius Center for Health Sciences and Primary Care Utrecht, University Medical Center Utrecht, Utrecht, The Netherlands; 20 PGOsupport, Dutch Networking Organisation for Patient Organisations, Utrecht, The Netherlands; 21 Department of Medical Humanities, Amsterdam UMC, Amsterdam, The Netherlands; 22 Department of Paediatric Rheumatology, University Medical Centre Utrecht, Wilhelmina Children’s Hospital, Room KC.03.063.0, P.O. box 85090, 3508 AB Utrecht, The Netherlands

**Keywords:** Juvenile Idiopathic Arthritis (JIA), Research agenda, James Lind Alliance, Patient involvement

## Abstract

**Background:**

Research on Juvenile Idiopathic Arthritis (JIA) should support patients, caregivers/parents (carers) and clinicians to make important decisions in the consulting room and eventually to improve the lives of patients with JIA. Thus far these end-users of JIA-research have rarely been involved in the prioritisation of future research.

**Main body:**

Dutch organisations of patients, carers and clinicians will collaboratively develop a research agenda for JIA, following the James Lind Alliance (JLA) methodology. In a ‘Priority Setting Partnership’ (PSP), they will gradually establish a top 10 list of the most important unanswered research questions for JIA. In this process the input from clinicians, patients and their carers will be equally valued. Additionally, focus groups will be organised to involve young people with JIA. The involvement of all contributors will be monitored and evaluated. In this manner, the project will contribute to the growing body of literature on how to involve young people in agenda setting in a meaningful way.

**Conclusion:**

A JIA research agenda established through the JLA method and thus co-created by patients, carers and clinicians will inform researchers and research funders about the most important research questions for JIA. This will lead to research that really matters.

## Background

Research priority setting involving the end-users of knowledge is clearly needed in order to formulate research questions that can really make a difference [1–3]. In a recent review, Odgers et al. reported a substantial increase in the number of research priority setting initiatives in paediatric chronic disease since 2010, generating a broad range of priorities shared across multiple conditions [4]. Unfortunately, the methodology was generally inadequately described. This lack of clarity raises concerns over the legitimacy and relevance of identified priorities. Odgers et al. suggested that the available systematic methods of priority setting should be used more often [4].

Patient and parent/caregiver (carer) involvement in establishing research priorities is crucial in generating a research agenda that encompasses the full spectrum of issues that affects paediatric patients with chronic disease [5–9]. Thus far, their involvement in research priority appears to be limited. Only approximately one-in-four studies reported some parental/caregiver involvement, and only 5% involved children directly [4]. Furthermore, qualitative research showed that the involvement of patients and carers seems to be challenging: real co-design does not happen by itself [3]. Therefore, precautionary measures need to be taken to empower patients and carers [7], and their involvement in the process should be monitored and evaluated [4, 10].

As noted above, understanding young people’s research priorities is crucial to develop research that is in tune with their needs [4, 11, 12]. However, some researchers have reported challenges to collaborating with young people in health research [12–14]. More recently, there appears to be increasing efforts to involve children and adolescents in research priority setting [7, 11, 12, 15]. Parsons et al. organised 13 focus groups involving young people with rheumatic diseases, aged 11–24, to explore what they believed to be important research questions regarding their condition [12, 16]. They provided evidence that even younger adolescents (11–15 years) are equipped to discuss and prioritise scientific research even if they are relatively research naive [16].

In 2018 four Dutch organisations of patients, parents and clinicians will start to establish a research agenda for Juvenile Idiopathic Arthritis (JIA). The research agenda was initiated by the Netherlands JIA patient and parent organisation (Dutch Juvenile Arthritis Association: DJAA), a member organisation of the European Network for Children with Arthritis (ENCA). Three other Dutch organisations are involved: the Dutch network organisation for young arthritis patients (16–30 years old) Youth-R-Well.com, the Dutch Association for Pediatric Rheumatology (DAPR) and the Dutch Health Professionals in Pediatric Rheumatology (DHPPR). The project will be based at the Pediatric Rheumatology and Immunology department of the Wilhelmina Children’s Hospital (WKZ) in Utrecht. DAPR, DJAA and WKZ funded the project. Amsterdam UMC will develop and lead the focus groups for young people with JIA, and enable them to have effective and fulfilling participation in the whole process. PGO-support, a Dutch networking organisation for patient organisations will issue an independent process evaluation of the PSP. The Department of Medical Ethics at the Julius Center of the UMC Utrecht will perform the research concerning the process evaluation.

In this paper, the project is outlined briefly. We discuss how we will address the aforementioned questions on the use of systematic methods and the feasibility of meaningful patient and caregiver participation in research agenda setting. Formulating a research agenda is not a goal in itself [2, 17]. Finally, we will describe how we aim to inspire researchers and research funders by the defined priorities when preparing for and guiding funding new research projects.

## Main text

In a recent review on patient and public engagement in priority setting, Manafo et al. described four highly structured deliberative methods that are inclusive and objectively based, specific to the priorities of all stakeholders engaged in the process [5]. Two of these methods have successfully been applied in the Netherlands: The Dialogue Method and the James Lind Alliance (JLA) Priority Setting Partnerships [18, 19]. Both methods are clearly suited for this purpose [5, 20]. Eventually, we chose the JLA method as it proved to be very effective in implementing agendas in calls for research. United Kingdom’s National Institute for Health Research (NIHR) and several funding charities, for example Marie Curie, the Multiple Sclerosis Society and Parkinson’s UK, have adopted the research agendas as an important part of their research funding strategy [21].

The James Lind Alliance (JLA) is a non-profit initiative established in the United Kingdom (UK) in 2004 by Sir Iain Chalmers [22]. The JLA team is positioned at the NIHR Evaluation, Trials and Studies Coordinating Centre (NETSCC), based at the University of Southampton in the UK. The goal of the JLA is to bring together the end users of scientific knowledge – patients, carers and clinicians – to jointly formulate a research agenda for a disease or type of care. In a so-called ‘Priority Setting Partnership’ (PSP) they gradually establish a top-10 list of the most important unanswered questions for their health area of interest [23].

Until now about 70 different Top 10s have been published. More information on the content and the background of these Top 10s can be found on the JLA website [24]. In 2015, Chalmers and colleagues compared the prioritised unanswered questions of several PSPs to the research questions they found in registered clinical trials. They demonstrated that the JLA method identifies questions and themes that are not yet being addressed in current studies [2].

The JLA method consists of 5 steps: setting up the steering group, gathering uncertainties, data processing and verifying uncertainties, interim priority setting, and final priority setting (see Table 1). In a free internet-based Guidebook the JLA method has been described in more detail [23]. Following these five steps, it takes approximately twelve to eighteen months to formulate a research agenda [23].

**Table 1:** The priority setting process in the James Lind Alliance methodology

Step in the process	Description
1. Setting up the steering group	A PSP is led by a steering group that coordinates the PSP and organises the activities. It will include representatives of patients, carers and clinicians.
2. Gathering uncertainties	An electronic survey questionnaire is distributed widely. Patients, carers and clinicians will be asked: “What questions would you like answered to improve the health and wellbeing of people with JIA?” For young people with JIA focus groups will be organised. Research recommendations stated in systematic reviews and clinical guidelines are searched for as well (i.c. the Dutch JIA-medication guideline and the European SHARE initiative).
3. Data processing and verifying uncertainties	Out-of-scope submissions are removed. The eligible submissions are categorised and rephrased as researchable questions. Duplicates and very similar submissions are combined. Questions that have already been answered in relevant good quality research will be removed.
4. Interim priority setting	The long list of in-scope verified uncertainties goes into an electronic interim priority setting survey. Patients, carers and clinicians are asked to choose (and possibly also rank) the 10 uncertainties from the list that are most important in their experience. Completed interim prioritisation results are grouped into patients, carers and clinicians, and separate scores kept to ensure fair weighting of the constituent groups. The top 25–30 questions are taken to the final workshop.
5. Final priority setting	In a final day-long workshop, 20–30 people (patients, carers and clinicians) discuss the questions and gradually agree on the final order of priority of the list, focusing especially on agreeing a ‘Top 10’. The Top 10 will be published on the JLA website, and in a peer reviewed journal.

A PSP is led by a steering group (10–15 people) that coordinates the PSP and organises the activities [23]. The Dutch JIA-PSP steering group will be led by a carer and a pediatric rheumatologist. The steering group will include representatives of patients (i.c. adult JIA-patients), carers (i.c. parents) and clinicians (i.c. pediatric rheumatologists, ophthalmologists, physiotherapists and nurses,). The four previously mentioned organisations recruited their members for the steering group. Four groups – pediatric rheumatologists, other health professionals, patients and parents – will be equally represented in the steering group. The two PSP Leaders made a selection for the pediatric rheumatologists, and invited one of the centers to invite one of their nurse practitioners, in order to achieve a balanced representation of clinicians of all 6 academic centres including their affiliated rehabilitation centre within the steering group. These decisions were made in collaboration with the chairs of both organisations. Furthermore, all Dutch centers for pediatric rheumatology will be represented in this steering group. A MD PhD-student coordinates the JIA-PSP, reviews the data collected, identifies the existing research evidence, and formulates potential research questions with input from the steering group. The PSP will be supported and guided by a trained JLA Adviser [23].

According to Odgers et al., the JLA approach may not be very well suited to children with chronic disease [4]. It demands creative and developmentally appropriate strategies to empower children to reflect on their situation, and how research could benefit them and to articulate their priorities [25, 26]. Inspired by the work of Parsons et al. we will organise additional focus group meetings involving children and adolescents with JIA. This established qualitative method allows the participants to draw from other participant’s knowledge and allows for a conversation among peers [25].

Specific themes and research questions formulated by these young patients will be added to our dataset of uncertainties from the survey. In the focus groups the young patients will discuss how they wish to be involved in the process and which arrangements need to be made in order for them to participate successfully. The results of the focus group will be discussed in the second meeting of the steering group. This may change the involvement of young patients in the process onwards.

Some precautionary measures to empower patients and carers are built into the JLA method. Patients and carers take part in the steering group. We will ensure that the chairing of the steering group is fair and neutral so as not to favour one group over another. The final priority setting workshop of the PSP is attended by patients and carers as well as clinicians and the opinions of all people at the workshop are valued equally [23]. The JLA supports an adapted Nominal Group Technique for PSPs choosing their priorities during the final workshop. One benefit of this technique is that it prevents the domination of discussion by a single person and encourages the participation of less assertive members. There is no hierarchy between the different participants; no one individual or group’s views or experiences are more valid than another’s. Nominal Group Technique is a well-established and well-documented approach to decision making. Despite these measures, engaging patients and carers in the complexities of health science based discussions of uncertainty is challenging [27]. To empower patients and carers in the steering group, most of them received a two-day training as a patient partner in research [28].

A process evaluation with a specific focus on the ethical aspects of the decision-making process is conducted parallel to the priority setting process [10, 27]. In the process evaluation, issued by PGOsupport and executed by a researcher from the Julius Center, attention will be paid to the inclusion of different stakeholders, their influence on the priority setting as well as facilitating and limiting factors for equal deliberation between the different stakeholders. For this, the researcher from the Julius Center will observe all key meetings during the project and perform additional interviews among the stakeholder groups. Important deliverables include evaluation of the suitability of the JLA method for patient organisations that aim to take a role as partner and/or driving force in scientific research; identification of critical success factors during the process in relation to patient representation; identification of specific requirements for appropriate involvement of children and adolescents. PGOsupport will disseminate the results of the evaluation via the relevant media in order to inform other patient organisations that consider a similar approach.

Formulating a research agenda for JIA is not a goal in itself. It is important that researchers and research funders are inspired by the defined priorities when preparing for and funding new research projects. Therefore all Dutch academic centers for pediatric rheumatology will be represented in the steering group. We will present our results at a 2019 meeting of a large Dutch/Canadian research project on personalised medicine, UCAN CAN-DU, funded by the Netherlands Organisation for Health Research and Development ZONMW, the Canadian Institutes of Health Research (CIHR) and Dutch Arthritis Foundation (ReumaNederland), to inspire further JIA-research. Funding agencies will be invited for our final workshop. Members and ambassadors of the Dutch JIA-PSP are involved in European organisations like ENCA, Paediatric Rheumatology European Society (PReS) and the European League Against Rheumatism (EULAR). Collectively, these measures will ensure the optimal implementation of the research agenda in different “layers” of research.

## Conclusions

In 2018 four Dutch organisations for JIA-patients, parents, pediatric rheumatologists and health professionals will initiate a PSP for JIA, following the JLA methodology. This research agenda, will be established in 2019, and will improve the relevance of JIA-research in the Netherlands and beyond. This will not only benefit patients, parents and clinicians in the consulting room, but also the JIA-researchers themselves since their research will really matter to the people that need it most.

## Abbreviations

CIHR: Canadian Institutes of Health Research
DAPR: Dutch Association for Pediatric Rheumatology
DHPPR: Dutch Health Professionals in Pediatric Rheumatology
DJAA: Dutch Juvenile Arthritis Association:
ENCA: European Network for Children with Arthritis
EULAR: European League Against Rheumatism
JIA: Juvenile Idiopathic Arthritis
JLA: James Lind Alliance
NETSCC: NIHR Evaluation, Trials and Studies Coordinating Centre
NIHR: National Institute for Health Research (NIHR)
PGOsupport: Dutch networking organisation for patient organisations
PReS: Paediatric Rheumatology European Society
PSP: Priority Setting Partnership
UCAN CAN-DU: Canada – Netherlands Personalized Medicine Network in Childhood Arthritis and Rheumatic diseases
WKZ: Wilhelmina Children’s Hospital
Youth-R-Well.com: Dutch network organisation for young arthritis patients (16–30 years old)
ZONMW: Netherlands Organisation for Health Research and Development
